# How a Facet of a Nanocrystal Is Formed: The Concept of the Symmetry Based Kinematic Theory

**DOI:** 10.1002/cphc.202200480

**Published:** 2022-11-09

**Authors:** Bing Ni, Guillermo González‐Rubio, Helmut Cölfen

**Affiliations:** ^1^ Physical Chemistry University of Konstanz Universitätsstrasse 10 78457 Konstanz Germany

**Keywords:** crystal growth, facet evolution, nanoparticles, non-faceted structure, symmetry based kinematic theory

## Abstract

Conventional nanocrystal (NC) growth mechanisms have overwhelmingly focused on the final exposed facets to explain shape evolution. However, how the final facets are formed from the initial nuclei or seeds, has not been specifically interrogated. In this concept paper, we would like to concentrate on this specific topic, and introduce the symmetry based kinematic theory (SBKT) to explain the formation and evolution of crystal facets. It is a crystallographic theory based on the classical crystal growth concepts developed to illustrate the shape evolution during the NC growth. The most important principles connecting the basic NC growth processes and morphology evolution are the preferential growth directions and the properties of kinematic waves. On the contrary, the final facets are just indications of how the crystal growth terminates, and their formation and evolution rely on the NC growth processes: surface nucleation and layer advancement. Accordingly, the SBKT could even be applied to situations where non‐faceted NCs such as spheres are formed.

## Graphical abstract

**Particle facet evolution** is proceeded by (1) the surface nucleation on top of the atomic layers of the existing nanocrystals, and (2) the layer advancement processes of all layers. The final facets are the indication of how the growth processes terminate. Meanwhile, the global structure (morphology) changes could be illustrated by the preferential growth directions.

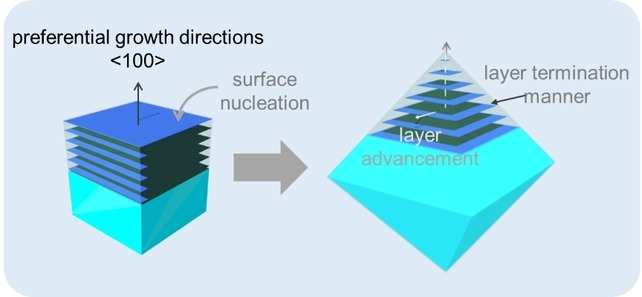

## 1 Introduction

The synthesis and fabrication of nanostructured materials have reached an unprecedented level of control after decades of unremitting efforts.[^1^, ^2^, ^3^, ^4^] The compositions of nanomaterials span from metals to alloys, metal oxides, chalcogenides, metal salts, high entropy materials, polymers etc. The morphology control has been mastered in many systems. Platonic polyhedra, concave or convex polyhedra enclosed by high index facets, hierarchical structures, etc., have been successfully prepared within many material systems via advanced synthesis routes. Furthermore, tight size control from the atomic level to sub‐nanometer scale, nanoscale, or even macroscale is also possible by using different fabrication strategies. Nowadays, the research topics in this field have been transferring to more intricate morphologies (such as chiral inorganic nanoparticles,[^5^, ^6^] subnanometric materials,^[7]^ mesocrystals,[^8^, ^9^] etc.), high‐throughput material discovering,^[10]^ controllable defected structures,^[11]^ finer size control and multi‐scale fabrications,[^12^, ^13^] and more complicated compositions,^[14]^ etc.

Meanwhile, the relationship between synthesis and applications is getting closer and closer. The synthesis is sometimes driven by possible applications. However, for the same nanocrystal (NC), the application focus is actually different from the synthesis focus. For example, heterogeneous catalysis focuses more on the facets of the NCs, and improving the mass activity of the catalyst generally relies on increasing the surface atom ratios.^[15]^ Instead, from a synthesis perspective, the bulk atoms, which account for a greater proportion, are the part that consumes more precursors, i. e., where growth plays a greater role, and the atoms on the surfaces simply indicate how the crystal growth terminates. However, these differences in focus have not been treated carefully, and the NCs are interrogated by their enclosed surfaces in most cases. The determining factor for the growth of different NCs is often considered to be crystal surface stability, also called thermodynamically‐controlled growth. Deviations would be categorized as kinetically‐controlled growth.^[16]^

However, this might not be enough for NC growth theory. For example for CaCO_3_, calcite is formed under thermodynamic control while vaterite is the kinetic product, which transfers to calcite with time and multiple kinetic processes could occur.^[17]^ If the growth processes themselves cannot be known, controlling the final morphologies would be quite challenging. To further excel the ability of NC morphology control and make the growth predictable and designable, deeper insights into growth at the atomic scale are highly required. Furthermore, there is actually a gap between the atomic scale growth events and the nanoscale morphology evolutions. The same atomic scale growth events would theoretically occur simultaneously at all the crystallographically equivalent sites. Therefore, understanding how the atomic scale growth events exert influence on the nanoscale morphology is also important for the precise synthesis of NCs.

The classical crystal growth theories have been widely explored in the past centuries^[18]^ (generally before the advent of nanochemistry[^19^, ^20^]). Although the experimental instruments didn't allow the atomic resolution analysis in those days, researchers proposed many theories by considering that the growth occurs via the atom‐by‐atom deposition manner. Through the comprehensive consideration of the Gibbs free energy changes, deposition‐dissolution equilibria, and mass diffusion, the basics of growth theories were established.^[21]^ The most widely known theory in this regard was proposed by Burton, Cabrera, and Frank, called the BCF theory.^[22]^ It suggests that the growth of a crystal relies on the 2D nucleation of a new atomic layer on the particle surface (formed by a bulk nucleation process that creates a new crystal phase) and the subsequent 2D layer advancement. Or in another way, the further growth can be controlled by the so‐called screw‐dislocation driven growth manner, which provides a non‐vanishing step site and bypasses the 2D nucleation process. If the crystal structure is considered, the Hartman‐Perdok theory which takes advantage of the periodic bond chains (PBCs) can be used to illustrate the growth kinetics.[^23^, ^24^, ^25^] Although many different theories have been proposed, it is generally agreed that the growth could be divided into three stages: nucleation, growth, and shape evolution. However, since the tracking of shape changes at the atomic scale or nanoscale was not realistic in those days and the importance of morphologies for properties was not realized, the shape evolution theories have not been adequately investigated. Only Frank borrowed some concepts from the theory of traffic flow on long crowded roads to establish a kinematic theory to theoretically address the shape evolutions in the 1970s.[^26^, ^27^] The theory mainly considers slow‐growing facets. The trajectory of the shape evolution can be predicted using a polar diagram of the slowness vectors (which is impossible to obtain experimentally). Although the theory is mathematically rigorous, the polar diagram is hardly suitable for predicting the shape evolution of real systems due to its complexity and the unavailability of the growth rates under experimental conditions.

Many modern mechanisms used to explain nucleation and growth of NCs have taken advantage of the classical theories. The most famous one is the LaMer model, which summarized a growth model of “instantaneous nucleation” followed by “diffusion‐controlled growth” based on the sulfur sol formation results in 1950.^[28]^ The model was actually not well recognized in the 20th century,^[29]^ but it is now widely used in illustrating the growth of monodisperse NCs. Furthermore, the delicate analysis of NC growth has also opened a way to the non‐classical growth theories. At the nucleation stage, theories like spinodal decomposition,^[30]^ pre‐nucleation clusters,^[31]^ coalescence of two separate amorphous sub‐nanometre clusters,^[32]^ etc., have been proposed and verified. As for the growth process, some special situations like oriented attachment and particle fusion have been found to defy the classical atom‐by‐atom growth manner.[^33^, ^34^] Nonetheless, the shape evolution theory has not been much advanced, and the final structures are usually attributed to the results of thermodynamically/kinetically‐controlled growth.

**Figure 1:**
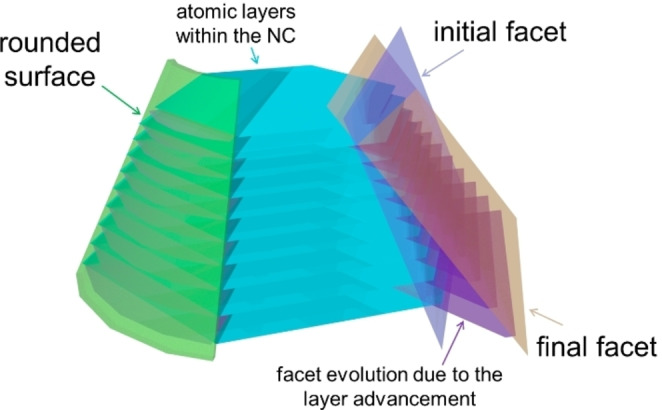
Simple illustration of the relationship between the atomic layers within the NC and the surface/facets

In this scenario, we recently proposed a symmetry‐based kinematic theory (SBKT) to illustrate shape evolution during NC growth.^[35]^ Notably, it is based on including the symmetry of the crystal lattice on the Frank kinematic theory, which greatly simplifies the analysis. Then we further introduced the concepts of the preferential growth directions (PGDs) and the properties of kinematic waves to illustrate shape evolutions in real cases. This has been recently well‐exemplified in a seed‐mediated growth of Au NCs. Here in this concept paper, we would like to focus on a specific aspect of crystal growth: how a facet of a NC evolves. Such an aspect of NC formation has not been properly tackled by the widely used thermodynamically/kinetically‐controlled growth mechanisms. The use of the SBKT to address it shall allow us to gain deeper insights into growth and shape evolution of NCs from the atomic scale. By considering the symmetry of the lattice and the NC shape, the growth at the atomic level can be correlated with the NC shape evolution at the nanoscale. According to the SBKT, the facet is not the most important aspect during crystal growth, it actually only indicates how the crystal growth terminates, i. e. how the layer advancement ends. If the atom layers terminate in a sharp manner, the NC would end up with a well‐defined facet. If the termination is in a gradual manner, the NC would end up with a rounded surface, and it would be difficult to define a facet (Figure 1). In the process of facet evolution, the already existing atom layers within the NC can start to expand again when a NC is put back into the growth environment, thus causing the evolution of the crystal faces, if there are still well‐defined crystal facets both before and after the secondary growth.

## 2 Conventional Facet Formation Mechanisms

As mentioned above, the formation of NC facets has not been specifically discussed previously. The conventional NC growth mechanisms usually convolute the facet formation and the morphology formation, i. e., a certain morphology could be obtained via the stabilization of the corresponding facets under the given growth conditions, which eventually defines the adopted shape. A good example for this is the Wulff construction for thermodynamically controlled growth.^[36]^ In most cases, the facet stabilization is investigated by theoretical calculations of NC surface free energy, which also allows to gain insights into the role of capping ligands as shape directing agents. In this context, we would like to present mechanisms commonly used to explain NC formation and shape evolution, and discuss their most significant limitations. To simplify the discussions, we would mainly focus on single crystalline Au NCs (i. e., defect‐free NCs) and some other noble metal NCs, based on the following reasons: (1) In many cases, control over their morphologies generally relies on seed‐mediated growth routes, where nucleation and growth stages occur in physical and temporal separated events. As a result, we don't need to take care of the bulk nucleation stage and only need to focus on the shape evolution occurring during the growth process; (2) their mechanisms have been extensively researched; (3) the NC only contains one element and the lattice is highly symmetric, which could further simplify the discussions. Several typical examples are shown here to illustrate the mechanisms.

**Figure 2:**
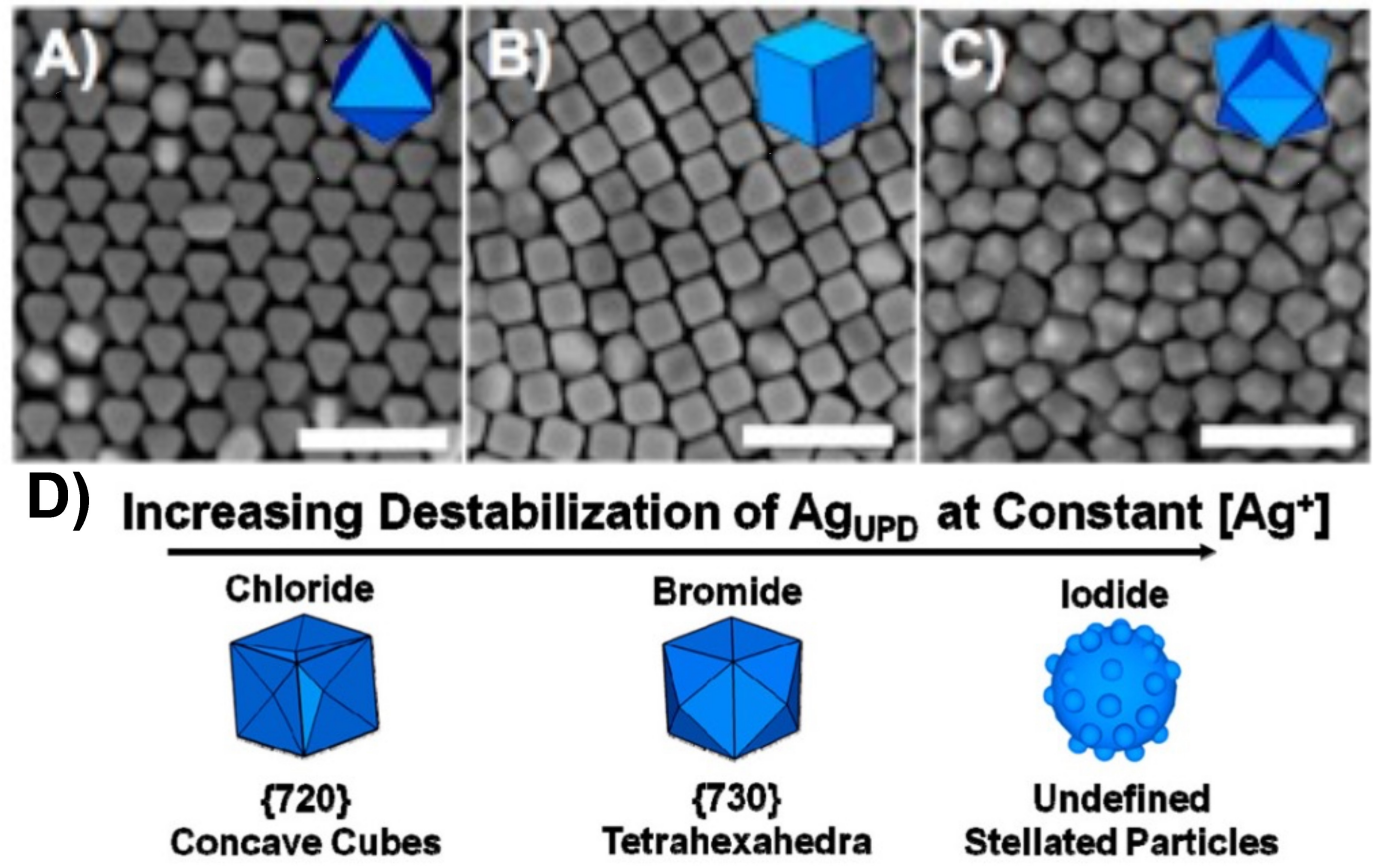
Au NC morphology changes with different concentrations of AA in the CTAB system. A) 0.5, B) 2.0, and C) 10.0 mM AA, respectively. Scale bars: 200 nm. D) The relationship between the stability of the Ag under potential deposition (Ag_UDP_) layers and the morphologies. Reproduced with permission from ref. [37]. Copyright 2012 American Chemical Society.

Illustrative examples of the growth of faceted Au NCs using seeded mediated growth routes utilized HAuCl_4_ as metal precursor and ascorbic acid (AA) as reducing agent, while quaternary ammonium surfactants are utilized as shape directing agents. The morphologies are typically adjusted by tuning the surfactant nature and the reactant concentrations. For instance, cetyltrimethylammonium bromide (CTAB) favors the formation of nanocubes, probably due to its ability to preferentially stabilize the {100} facets.[^38^, ^39^, ^40^, ^41^] Therefore, Au nanocubes should be the main product in those cases where CTAB passivates {100} facets. On one hand, this has been achieved at proper reactant concentrations. On the other hand, it was also found that different shapes could be obtained by only changing the concentration of AA (Figure 2A–C).^[37]^ When the AA concentration increased, the final Au NC morphologies would transit from octahedra, to cubes and trisoctahedra. According conventional growth mechanisms, this effect could be ascribed to an increased rate of atom deposition when using higher AA concentrations, which favor the formation of high index facets (the deposited atoms do not have time to diffuse to a thermodynamically more favored site before the deposition of another atom on the NC surface occurs). In such systems, CTAB controls the formation of thermodynamic products while AA determines the kinetically favored ones. However, since the other reactant concentrations have not been changed, the surface stabilization effect of CTAB shouldn't change. Therefore, it would be necessary to understand under which circumstances the growth can be switched from the thermodynamic products to the kinetic products. Nevertheless, this has not been unambiguously answered.

**Figure 3:**
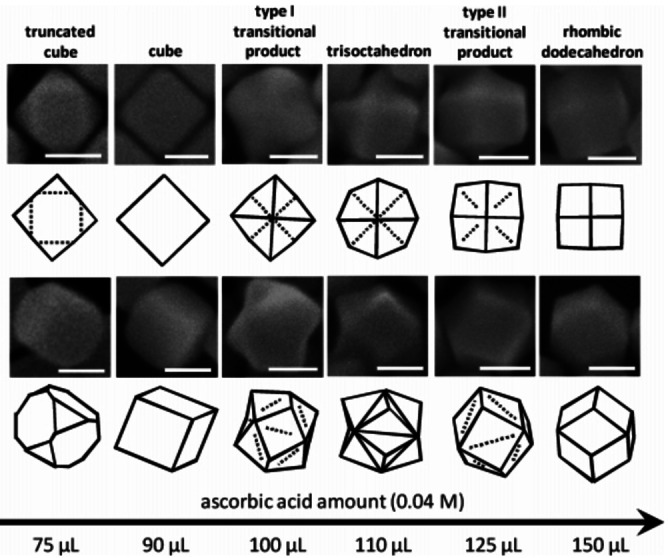
Au NC morphology changes with different amounts of AA additions in the CTAC system. Scale bars: 50 nm. Reproduced with permission from ref. [43]. Copyright 2010 American Chemical Society.

The phenomena were also found in other cases. For instance partial replacement of bromide with chloride as counterions strongly impact the interaction of the CTA^+^ (CTAC) micelles with gold surfaces (less dense micellar layers^[42]^), leading to the formation of distinct faceted NCs when increasing AA concentration (Figure 3).^[43]^ Another interesting different growth effect in such system is that CTAB can produce {730}‐facet‐enclosed convex tetrahexahedra,^[44]^ while CTAC favors {720}‐facet‐enclosed concave cubes at similar conditions (Figure 2D).^[45]^ The difference between {730} facets and {720} facets is quite small, but the convex and concave morphologies are quite different. This is also to say that the surface stabilization effect cannot explain the difference between convex and concave shapes. The authors suggested that the formation of concave and convex shapes was due to the stability of the Ag under potential deposition (Ag_UDP_) layers (Ag ions were added in the synthesis in these cases). The presence of Cl^−^ could kinetically trap or lock the NC into a particular faceted structure in the early growth stage, while the Br^−^ could better stabilize the Ag_UDP_ layers, thereby leading to distinct shape evolutions. When using cetylpyridinium chloride (CPC) as the surfactant, it should more stabilize the {111} facets (i. e., octahedron should be obtained), while the existence of Br^−^ in the system could alter the shape to cubes which means {100} facets are more preferred.^[46]^ However, seemingly contradictory results were also found in these systems, of which more Br^−^ ended in the increased exposition of {111} facets (from truncated cube to truncated octahedron).^[47]^

The surface stabilization effect encountered many problems in illustrating the growth mechanisms. To address the aforementioned deviations, a category of kinetically‐controlled growth was created.^[48]^ For example, the formation of Au cubes in the CTAB system can be seen as the stabilization of {100} facets, therefore it is a kind of thermodynamically‐controlled growth, and Au octahedra and trisoctahedra are the results of kinetically‐controlled growth. In such systems, CTAB controls the formation of thermodynamic products while AA determines the kinetically favored ones. However, the line between the kinetically‐controlled growth and the surface stabilization (or the thermodynamically‐controlled growth) cannot be clearly drawn, and their physical difference has not been unambiguously depicted, probably due to the difficulties in disentangling the effects exerted by the different reactants and capping agents on both, the growth kinetics and the thermodynamic stability of the NCs. For instance, the use of CTAB or CTAC affects the facet stabilization, thereby impacting the thermodynamics of the system. However, the nature of the halide also impacts the reduction potential of gold ions, as they are in the form of AuX_4_^−^. In the presence of Cl^−^, the reduction of gold precursor is favored, which enhances the kinetics of NC growth (facilitates Au atom deposition).[^37^, ^49^] In this context, questions like how fast is fast enough to drive the growth to the kinetically‐controlled growth and what kind of deviated structures should be formed, would arise if one wants to understand NC growth based on kinetic and thermodynamic concepts. The key reason for the inability of the existing growth models is that they ignore the basic crystal growth processes, i. e., how the atoms in precursors become the entity of the NCs, and how these processes could be linked with the global structure changes at the nanoscale. Younan Xia then proposed a deposition‐diffusion growth manner^[16]^ to address this problem, which suggested that the atoms would firstly deposit at the corners and then diffuse to other regions. The relative rates of atom deposition and surface diffusion could determine the final morphology. When the deposition is faster than the surface diffusion, branched structures would be formed (kinetically‐controlled growth), while well‐faceted structures could be obtained at faster diffusion rates and the facets depend on the diffusion rates (thermodynamically‐controlled growth). Although this model has been used in many cases, more questions arose within this model, for example, why the atoms prefer to deposit at the corners, what is the driving force for the surface diffusion and how to tune it, how general this model is and what the reason is if this model can be applied in different systems, etc. Furthermore, this model cannot nicely explain the symmetry‐breaking growth to form nanorods, for example, and what the difference between a cube and a cuboid is from the growth perspective.

Therefore, more attempts are needed to address the NC growth mechanisms. A crystallographic theory that doesn't rely on specific materials should be highly appreciated in this regard. Although BCF theory was successful to illustrate the basic crystal growth process, it can only help to explain the shape evolutions when the screw dislocation is responsible for the morphologies. In this scenario, the formation of certain spiral structures, nanowires, and nanotubes can be explained according to the supersaturations of the precursors and the strength of the screw dislocation strain effects.[^50^, ^51^, ^52^] Another type of thermodynamically‐controlled growth would be the formation of Wulff structures which are mainly the cuboctahedron or truncated shapes. The Wulff construction illustrates the equilibrium shape of a particle by considering that, in equilibrium, the distances of the crystal facets from a point within the crystal are proportional to the corresponding specific surface energies of these faces. This theory mainly relies on the theoretical calculation of the surface energies, and can only deal with equilibrium shapes. Therefore, its use is also limited. Frank proposed a kinematic theory to explain the theoretical shape evolutions of any crystal (named as Frank's kinematic theory or classical kinematic theory).[^21^, ^26^] In the following sections, we will explain the concepts and limits of the theory, as well as our modifications, which make the theory applicable.

## 3 The Symmetry Based Kinematic Theory (SBKT)

With the development of SBKT, we aim at addressing the phenomenon of shape evolution during NC growth, which in fact is a process that depends on several phenomena occurring at different scales. Thus, the whole conceptual hierarchies in the SBKT would be:^[35]^

Level 1: shape evolution
Level 2: preferential growth directions (PGDs), kinematic waves
Level 3: surface nucleation, layer advancement
Level 4: atom deposition, surface atom diffusion

The concepts at level 3 and level 4 are the basic atomic growth mechanisms and have been carefully discussed in the BCF theory.^[22]^ These events occur at the atomic scale, and the same events would theoretically occur simultaneously at all the crystallographically equivalent sites, convoluting together to drive the shape evolutions. To link these events with nanoscale morphology evolutions (level 1), concepts at level 2 are needed. Since we mainly focus on the shape evolutions, the concepts at level 3 and 4 will not be discussed in detail here.

**Figure 4:**
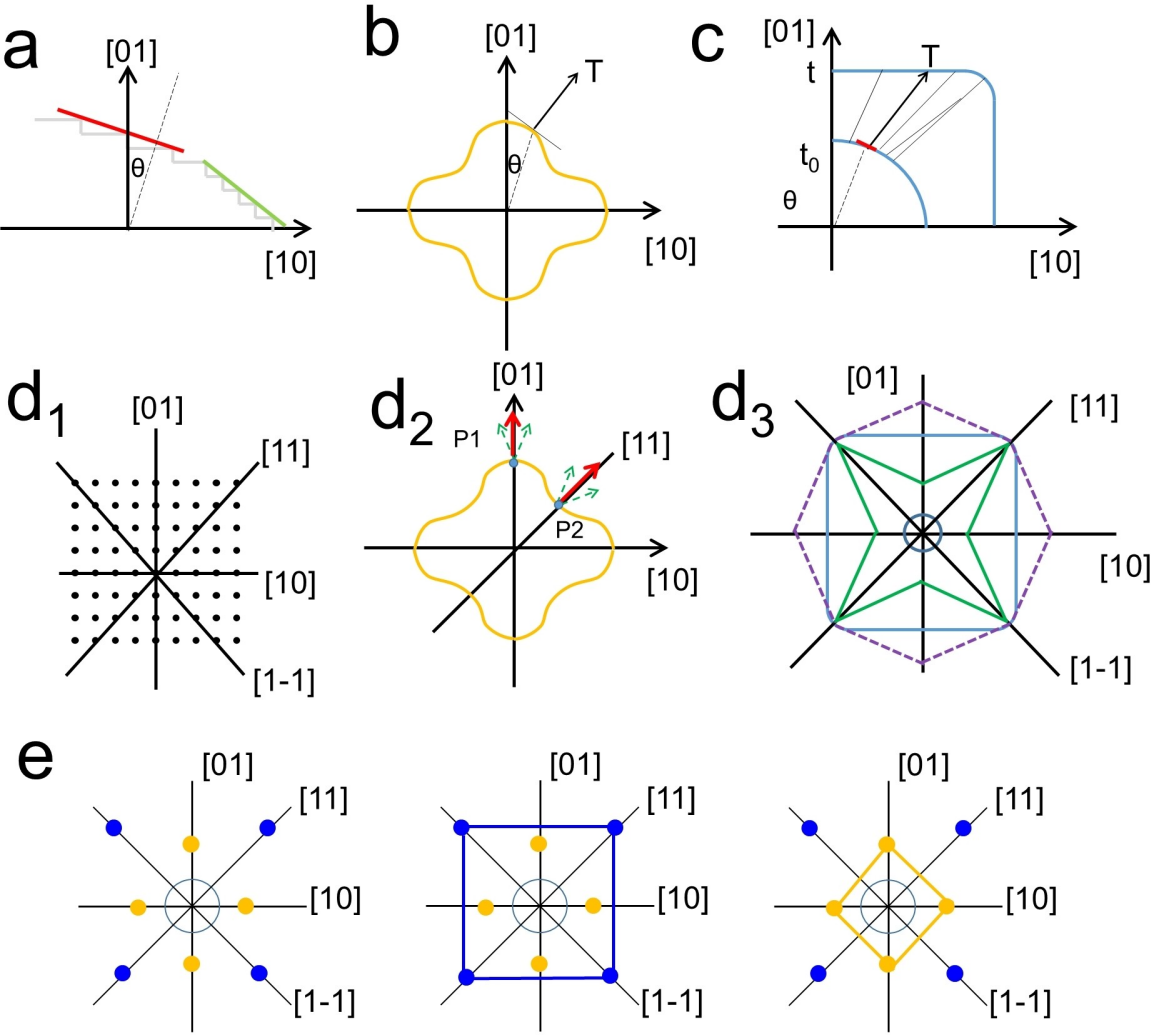
a)–c) Geometric expression of Frank's kinematic theory. The crystal is put into a virtual coordination system constructed according to the lattice (a). Then the facet of interest (red line) is marked; a hypothetical slowness vector polar diagram (b); the morphology evolution from the arc (t_0_) to the square (t) according to the polar diagram (c). d) the development of the SBKT: (d1) a hypothetical two‐dimensional simple square lattice, and the mirror planes are labeled; (d2) according to the mirror symmetry analysis, the normal of P1 and P2 in the slowness vector polar diagram must be along the [01] and [11] directions, respectively; (d3) the shape evolution from the inset circle to the square or star‐like structure could be obtained by connecting the equivalent points at the <10> and <11> directions, respectively. Reproduced with permission from ref. [35]. Copyright 2022 Wiley‐VCH. e) the details of how to construct the final shapes by connecting the corresponding equivalent points at the directions when high index facets are not considered.

To demonstrate the SBKT, we would like to briefly introduce the Frank's kinematic theory first.[^21^, ^26^] The geometric expression of the theory is shown in Figure 4a–c. To evaluate the evolution of a facet (red line), the normal of this facet needs to be labeled, as well as the angle θ, which are considered in a virtual coordination system based on the lattice structure (any square, tetragonal or orthogonal lattice in this exemplary case, where perpendicular axes are [01] and [10], Figure 4a). Then θ is used to find the point of intersection in the slowness vector polar diagram (constructed by plotting the reciprocal of the growth velocities in different directions) that corresponds to the given facet (red line in Figure 4a), and, at this point, the corresponding normal T to the tangent at that point indicates the evolution direction of the facet (Figure 4b). It is worth mentioning that the normal T typically does not coincide with the normal of the facet (red line in Figure 4a). By doing this operation with all the vicinal facets at the initial structures, the shape evolution trajectory could be obtained in real space (for example, from a circle to a square, Figure 4c). It can be seen that this theory is very complicated and presents some key issues that impair its practical implementation for analysis of real cases. The most relevant is that the slowness vector polar diagram is impossible to experimentally or theoretically obtain, which eventually makes the 3D application of this theory, which is relevant in most of the real syntheses, extremely challenging.

To make the Frank's kinematic theory practical, we introduced the lattice symmetry into the theory. Like traditional crystal growth researchers prefer to use a hypothetical Kossel crystal (a crystal with a simple cubic lattice, which does not exist in nature) to develop the theory, we also developed the SBKT from a hypothetical two‐dimensional simple square lattice (Figure 4d1). Therefore, the symmetry elements here are *C_4_*, *m*, $\bar{1}$ . Accordingly, the polar diagram must contain the same symmetry elements. The mirror planes lie along with the <10> and <11> directions. Their intersections with the polar diagram are labeled as P1 and P2. Consequently, the normals of P1 and P2 at the polar diagram must be along with the [01] and [11] directions, respectively. Otherwise, there would be two normals of one point (green arrows) according to mirror symmetry, which is impossible (Figure 4d2). This suggests that the evolution trajectory of {10} and {11} facets would be always along their facet normal. Another conclusion that can be made from this mirror symmetry and other symmetry elements is that the P1 and P2 must represent the local minimal or maximal growth speed in the polar diagram (if this would not be the case, the two sides of the P1 or P2 would have a smaller value and the other a larger value, which contradicts the mirror symmetry). Therefore, even though the polar diagram is not known, (1) the growth trajectories of the {01} and {11} facets will always be along their normals, respectively; (2) their growth speed must be the local minimum or maximum. To illustrate the shape evolution trajectory, the NC can be put into a virtual coordination system, and these <01> and <11> directions can be plotted, which are actually the growth trajectories of the corresponding facets. The final shapes could be obtained just by connecting the corresponding equivalent points at the directions (Figure 4e). If the high index facets cannot exist, a square would be obtained at the end (Figure 4e). The exposed facets depend on the relative growth rate of <01> and <11> directions. If <01> directions grow fast, the facet would eventually disappear and the final structure exposes {11} facets, and *vice versa*. If high index facets can be obtained under the given growth conditions, concave or convex star‐like shapes could be obtained depending on the relative growth rates of <01> and <11> directions (Figure 4d3). This analysis also showed that whether the high index facets are formed or not can be predicted from the relative growth speed along <01> and <11> directions according to the polar diagram on the other hand. However, since the polar diagram is inaccessible, we would like to consider the situation of high index facets as an input for the further analysis (a yes or no question without the need to know the exact index of the high‐index facets). Based on these deductions, the theory can be further simplified by just considering one set of the fast growth directions along the mirror planes, or in other words the preferential growth directions (PGDs), to illustrate the shape evolutions. The PGDs are the directions along the mirror planes that grow the fastest (see further discussion 1 in the supporting information, SI). The growth trajectories of the facets perpendicular to the PGDs would be always along the PGDs, and eventually disappear. Altogether, by simply considering the PGDs and the lattice symmetry, the SBKT contains all the content in Frank's kinematic theory. Once the PGDs are known, the growth trajectory could be depicted. The same can be extended to other lattices besides the simple square lattice (see further discussion 2 in the SI).

So far, we have simplified Frank's kinematic theory. But one can notice that we obtain the final shape just by connecting the corresponding equivalent points at the PGDs, the atomic viewpoints of facet formations or evolutions have not been discussed. On one hand, the PGDs are used to define the evolution trajectories. Additionally, since the PGDs reflect the lattice symmetry, the final NC morphology would typically feature the same symmetry as the lattice symmetry.

On the other hand, the kinematic wave holds the key to forming the facet. The kinematic wave is the collective behavior of the layer advancement processes. The detailed mathematical forms of the kinematic wave are not needed to illustrate the shape formations. It is more important to know the properties of the kinematic wave (Figure S1): (1) the atomic non‐uniformities on the surface of NCs would propagate to other regions during growth (the mathematical forms of this movement can be treated as waves, this is why the movement is called kinematic wave); (2) after sufficiently long growth, the kinematic wave will dissipate, i. e., the atomic non‐uniformities will disappear; (3) kinematic waves at different regions of the NC could interact once they meet at a point. The proof of these properties can be found in our recent report.^[35]^ The next question would be what an atomic non‐uniformity during the growth is. If the slowness vector polar diagram could be known, the answer could directly be obtained. However, since the polar diagram is inaccessible, we would like to explore the answer from the real growth conditions. If the growth conditions do not prefer the formation of high index facets, the final facets could be depicted by connecting the corresponding equivalent points at the PGDs. Therefore, any surfaces/facets that deviated from those would be the non‐uniformities. If high index facets can be formed under the given conditions (no matter which high index facet), the most reasonable shape should have the same symmetry as the lattice symmetry, therefore, any protrusions or surface structures that might harm the NC symmetry are the non‐uniformities (Figure S2). Due to the propagation and dissipation properties of the kinematic waves, we can deduce the features of the facet during the growth: (1) If the growth is sufficiently long, the final exposed facets would be the same due to the full dissipation of atomic non‐uniformities. Otherwise, building blocks would prefer to deposit on facets with higher reactivity (atomic non‐uniformity in other words), eventually leading to their vanishing. (2) If the amount of building blocks is not sufficient to dissipate all non‐uniformities of the structure, intermediate structures could then be obtained.

**Figure 5:**
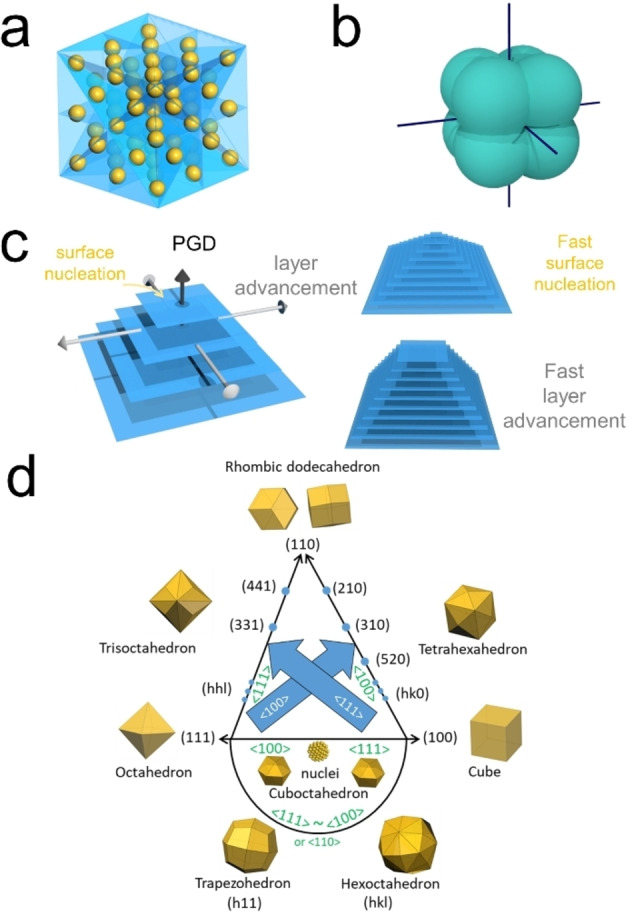
a) All the mirror planes in the symmetry elements of the fcc lattice (cF Bravais lattice) have been marked by the blue planes. It can be noticed that the intersections of the mirror planes are along the <111>, <110>, and <100> directions. Since we just considered the mirror planes of the lattice here, the same analysis could be extended to any cubic, tetragonal, and orthorhombic Bravais lattice (cP, cI, cF, tP, tI, oP, oS, oI, oF). Similar mirror symmetry analysis can also be done with the hexagonal Bravais lattices. b) a hypothetical slowness vector polar diagram is drawn here to show its symmetry according to the lattice symmetry. (c) schematic illustrations of the effects of fast surface nucleation or layer advancement. (d) Shapes that could be obtained by connecting the corresponding equivalent points at the <111>, <110>, and <100> directions. See details at Figure S3. Reproduced with permission from ref [35]. Copyright 2022 Wiley‐VCH.

**Figure 6:**
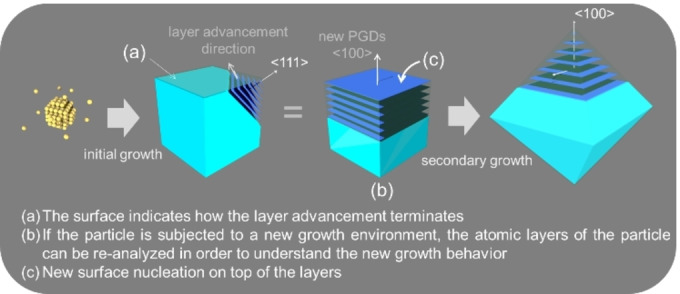
Schematic illustration of the facet evolutions when the NC is subjected to a new growth environment. It is worth mentioning that the same growth process (surface nucleation, layer advancement) would occur at all the crystallographic equivalent positions at the same time, leading to the global structure changes.

Now we will extend the discussion to the 3D cases. For the sake of simplicity, we will discuss the fcc lattice here. When the mirror planes are labeled in the lattice, it could be found that the intersections of mirror planes are along the <111>, <110>, and <100> directions (Figure 5a). Similar to the symmetry analysis shown in Figures 4d1 and 4d2, it can be concluded that the growth trajectories of the {111}, {110}, and {100} facets will always be along their normals, respectively (Figure 5b); (2) their growth speed must be the local minimum or maximum; (3) one set of these directions could be considered as the PGDs. That is to say the PGDs should be one set of the <111>, <110> and <100> directions. PGDs are where the growth is fast. The growth includes two aspects: surface nucleation and layer advancement (Figure 5c). Faster surface nucleation would lead to a pyramid structure, while faster layer advancement would result in a small terrace at the growth front. Nonetheless, the areas of facets perpendicular to the PGDs would minimize after sufficient growth. If we consider this effect from the perspective of surface energies, that is to say the surface with the highest energy would be the facet perpendicular to the PGDs. Once the PGDs are determined, similar growth trajectories shown at Figure 4d3 and the corresponding morphologies could be achieved (Figure 5d, Figure S3). It is worth mentioning that all the shapes shown in Figure 5d have been experimentally obtained.[^37^, ^53^] As for the properties of kinematic waves to construct the final facets, the conclusions are the same as those discussed with a simple square lattice. Once these factors are known, the experimental researchers could determine the final morphologies that could be obtained with this growth system after optimizations. Retrospecting the shape formation according to the SBKT, the shape of the NCs is defined by the PGDs and properties of kinematic waves (concepts at level 2), and the facets just indicate how the crystal growth terminates, i. e. how the growth processes like surface nucleation and layer advancement end (concepts at level 3). If the atom layers terminate in a sharp manner, the NC would end up with a well‐defined facet. If the termination is in a gradual manner, the NC would end up with a rounded surface, and it would be difficult to define a facet (Figure 1). When the seeds are subjected to a new growth condition, the facet evolutions are validated by the surface nucleation and layer advancement processes under the new growth conditions (Figure 6). Once these growth processes end, a new facet/surface different from the initial ones would be obtained.

Altogether, the lattice symmetry, PGDs, and high index facets are the three most important factors to determine the growth trajectory, as well as the final shapes and facets. These factors can be obtained by simple pre‐experiments. (1) The lattice symmetry: it can be known once the phase of the product is known. If twin boundaries exist in the products, the symmetry needs to be modified accordingly. Additionally, the formation of twin boundaries would also change the surface nucleation behavior,^[54]^ which could also complicate the properties of the kinematic waves. Therefore, we would not include the discussion of twinned NCs here. (2) PGDs: besides the surface energy calculations, the NCs in the pre‐experiments can also clearly reveal the PGDs, as well as whether high index facets can be formed, even though the experimental conditions are not optimized. Since PGDs are located where the growth is fast, the facets perpendicular to the PGDs would disappear. Therefore, the directions pointing to the corners of the NCs should be the possible PGDs. If there exist several different corner directions, the crystallographic symmetry would give a hint about the real PGDs. In very few cases, directions pointing to the edge centers might also be the PGDs, such as tetrahedral structures of a fcc lattice,^[55]^ anisotropic structures like nanorods.^[35]^ Overall, the PGDs and the properties of kinematic waves are dependent on the growth conditions. (3) To check if high index facets could exist, this could be easily seen from the morphologies of the NCs in the pre‐experiments. For example, when considering the growth of fcc latticed materials, if the morphologies are cubes, octahedral, or their truncated structures, and no rounded structures are found, the high index facets would not exist under the given growth conditions; if rounded structures are widely founded, the high index facets would exist under the given growth conditions. Ag NCs are typical examples that refuse the formation of high index facets,[^56^, ^57^] while Au NCs are the opposite.^[37]^ If we consider the formation of high index facets from the perspective of growth speeds (or the slowness vector polar diagram), it can be deducted that their formation can be predicted from the relative speeds along the directions (Figure 4d3). However, since the polar diagram cannot be obtained, it would be more convenient to use the situation of high index facets as the input. In some cases, the PGDs and high index facets remain robust even though the growth conditions have changed a lot.^[35]^

With this in mind, we can use the SBKT to analyze the reported results. From the kinematic viewpoint, increasing the concentration of AA could change the growth speeds of Au NCs along all possible directions. Figure 2 showed that the PGDs would shift from <100> directions (Figure 2A) to <111> directions (Figure 2B, C) when the AA was increased. The difference between Figure 2B and 2 C is due to the completion of growth. When the consumption of the Au precursor is not extremely fast, cubes would be the main product. However, when the Au precursor is consumed in a very short time due to the very high AA concentrations, trisoctahedra could be obtained since there are not enough Au atoms to fully smooth the tip protrusions (formed by rapid surface nucleation processes) to form a flat cube structure (Figure S4). The same analysis could be adopted to the CTAC system (Figure 3, S5) and the CPC system. As for the difference between the CTAB and CTAC system to produce convex and concave particles, it can also be easily explained by the PGDs of <100> (convex shape) and <111> (concave shape) directions, and in both cases the high index facets are maintained (Figure S4). The SBKT could also rationalize some reported results adopting growth directions to illustrate the morphology changes, such as the reported case of evolution from Ag cubes to dodecahedra and trisoctahedra^[58]^ (Figure S6). Since the facet evolution just relies on the PGDs and properties of the kinematic waves of the new growth condition, different faceted seeds should evolve to the same final structures after sufficient growth.^[59]^

**Figure 7:**
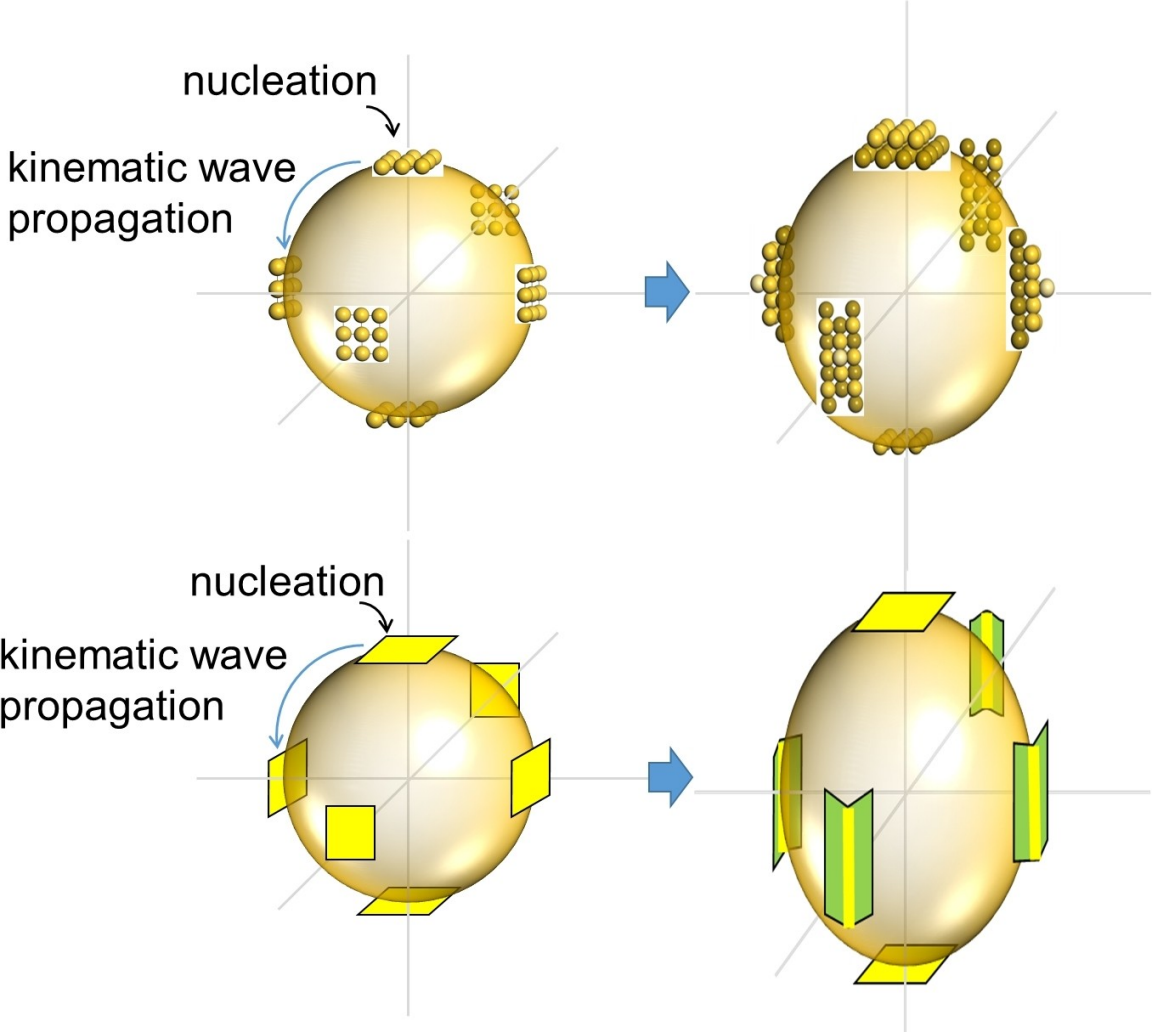
Symmetry breaking growth due to the coherent growth. The atomic structures in the upper panel indicate the nucleation sites. The nucleation sites in the middle areas would be changed due to the kinematic wave from the top nucleation site. Therefore, the further growth at the middle sites and the top/bottom sites would be different. The yellow planes in the lower panel indicate the size of the nucleation sites to show the symmetry breaking. Reproduced with permission from ref [35]. Copyright 2022 Wiley‐VCH.

The SBKT could even be used to explain the symmetry breaking growth (Figure 7). The main reason is attributed to a so‐called coherent growth: (1) the kinematic wave initiated due to a nucleation event at a nucleation site could propagate to the adjacent nucleation sites and therefore change the atomic structures of the adjacent nucleation sites; (2) meanwhile, a new nucleation event is going to occur before the kinematic wave has propagated through the NC to dissipate all the non‐uniformities. Before this process, all the nucleation sites are equal, thus, the speeds of surface nucleation and layer advancements are the same, the symmetry breaking growth would not occur. However, once the nucleation sites become non equal, the speeds of surface nucleation at non‐equal nucleation sites would become non equal as well. Therefore, symmetry breaking growth would occur as is for example evident in Au‐nanorod growth.[^35^, ^44^, ^60^]

## 4 Summary and Outlook

The SBKT provides a new crystallographic theory to interrogate the NC shape formation. It jumps out of the shackles of facets. Instead, it mainly uses PGDs and the properties of the kinematic waves to illustrate the shape formation. Therefore, the SBKT could be also used for structures without well‐defined facets, which can hardly be included in other conventional NC formation mechanisms. The facets just indicate how the growth ends. For example, when the growth speeds along different directions are similar and the amount of precursors is not sufficient, then spherical particles would be formed. If the NCs are subjected to a new growth condition, the initial facets would evolve to other facets or rounded surfaces according to the growth conditions. The SBKT shows a way to control the final morphologies by just tuning the growth kinetics. It reconciled the previously categorized thermodynamically/kinetically‐controlled growth. If the coherent growth has not occurred and the precursor amount is sufficient enough, isotropic structures could be obtained, which could be seen as the thermodynamically‐controlled growth. Otherwise, the products could be seen as the kinetically‐controlled growth.

The limitation of the SBKT arises from the limits of the classical crystal growth theories. It cannot be used in the shape evolutions of non‐classical processes, such as oriented attachment,^[33]^ particle fusion,^[34]^ and structural changes in dynamic processes far from equilibrium,[^52^, ^61^] etc. Since most of these non‐classical processes occur based on the already formed NCs, these processes can be combined with the SBKT to extend our full ability to design nano morphologies. Once the understanding of non‐classical processes has been perfected, it might be possible to achieve the total synthesis of inorganic materials like those of the total synthesis of natural organic products

## Conflict of interest

The authors declare no conflict of interest.

5

## Supporting information

As a service to our authors and readers, this journal provides supporting information supplied by the authors. Such materials are peer reviewed and may be re‐organized for online delivery, but are not copy‐edited or typeset. Technical support issues arising from supporting information (other than missing files) should be addressed to the authors.

Supporting Information

## Data Availability

The data that support the findings of this study are available from the corresponding author upon reasonable request.

## References

[1] Y. Xia , Y. Xiong , B. Lim , S. E. Skrabalak , Angew. Chem. Int. Ed. 2009, 48, 60–103; 10.1002/anie.200802248 PMC2791829 19053095

[2] K. D. Gilroy , A. Ruditskiy , H.-C. Peng , D. Qin , Y. Xia , Chem. Rev. 2016, 116, 10414–10472. 27367000 10.1021/acs.chemrev.6b00211

[3] J. J. Calvin , A. S. Brewer , A. P. Alivisatos , Nat. Synth. 2022, 1, 127–137.

[4] M. J. Banholzer , J. E. Millstone , L. Qin , C. A. Mirkin , Chem. Soc. Rev. 2008, 37, 885–897. 18443674 10.1039/b710915f PMC8207723

[5] H.-E. Lee , H.-Y. Ahn , J. Mun , Y. Y. Lee , M. Kim , N. H. Cho , K. Chang , W. S. Kim , J. Rho , K. T. Nam , Nature 2018, 556, 360–365. 29670265 10.1038/s41586-018-0034-1

[6] G. González-Rubio , J. Mosquera , V. Kumar , A. Pedrazo-Tardajos , P. Llombart , D. M. Solís , I. Lobato , E. G. Noya , A. Guerrero-Martínez , J. M. Taboada , F. Obelleiro , L. G. MacDowell , S. Bals , L. M. Liz-Marzán , Science 2020, 368, 1472–1477. 32587018 10.1126/science.aba0980

[7] B. Ni , Y. Shi , X. Wang , Adv. Mater. 2018, 30, 1802031. 10.1002/adma.201802031 30039573

[8] C. Jenewein , J. Avaro , C. Appel , M. Liebi , H. Cölfen , Angew. Chem. Int. Ed. 2022, 61, e202112461. 10.1002/anie.202112461 PMC9298807 34669241

[9] H. Cölfen , M. Antonietti , Angew. Chem. Int. Ed. 2005, 44, 5576–5591; 10.1002/anie.200500496 16035009

[10] J. C. Dahl , X. Wang , X. Huang , E. M. Chan , A. P. Alivisatos , J. Am. Chem. Soc. 2020, 142, 11915–11926. 32531162 10.1021/jacs.0c04997

[11] M. H. Oh , M. G. Cho , D. Y. Chung , I. Park , Y. P. Kwon , C. Ophus , D. Kim , M. G. Kim , B. Jeong , X. W. Gu , J. Jo , J. M. Yoo , J. Hong , S. McMains , K. Kang , Y.-E. Sung , A. P. Alivisatos , T. Hyeon , Nature 2020, 577, 359–363. 31942056 10.1038/s41586-019-1899-3

[12] G. González-Rubio , V. Kumar , P. Llombart , P. Díaz-Núñez , E. Bladt , T. Altantzis , S. Bals , O. Peña-Rodríguez , E. G. Noya , L. G. MacDowell , A. Guerrero-Martínez , L. M. Liz-Marzán , ACS Nano 2019, 13, 4424–4435. 30939242 10.1021/acsnano.8b09658

[13] L.-B. Mao , H.-L. Gao , H.-B. Yao , L. Liu , H. Cölfen , G. Liu , S.-M. Chen , S.-K. Li , Y.-X. Yan , Y.-Y. Liu , S.-H. Yu , Science 2016, 354, 107–110. 27540008 10.1126/science.aaf8991

[14] E. P. George , D. Raabe , R. O. Ritchie , Nat. Rev. Mater. 2019, 4, 515–534.

[15] Y. Shi , Z. Lyu , M. Zhao , R. Chen , Q. N. Nguyen , Y. Xia , Chem. Rev. 2021, 121, 649–735. 32667792 10.1021/acs.chemrev.0c00454

[16] Y. Xia , X. Xia , H.-C. Peng , J. Am. Chem. Soc. 2015, 137, 7947–7966. 26020837 10.1021/jacs.5b04641

[17] H. Cölfen , S. Mann , Angew. Chem. Int. Ed. 2003, 42, 2350–2365; 10.1002/anie.200200562 12783497

[18] S. Karthika , T. K. Radhakrishnan , P. Kalaichelvi , Cryst. Growth Des. 2016, 16, 6663–6681.

[19] G. A. Ozin , Adv. Mater. 1992, 4, 612–649.

[20] M. Whitesides George , P. Mathias John , T. Seto Christopher , Science 1991, 254, 1312–1319. 1962191 10.1126/science.1962191

[21] I. V. Markov , Crystal growth for beginners: fundamentals of nucleation, crystal growth and epitaxy, World scientific, 2016.

[22] W. K. Burton , N. Cabrera , F. C. Frank , N. F. Mott , Philos. Trans. R. Soc. London Ser. A 1951, 243, 299–358.

[23] P. Hartman , W. Perdok , Acta Crystallogr. 1955, 8, 49–52.

[24] P. Hartman , W. Perdok , Acta Crystallogr. 1955, 8, 521–524.

[25] P. Hartman , W. Perdok , Acta Crystallogr. 1955, 8, 525–529.

[26] F. Frank , Z. Phys. Chem. 1972, 77, 84–92.

[27] M. J. Lighthill , G. B. Whitham , Proc. R. Soc. London Ser. A 1955, 229, 317–345.

[28] V. K. LaMer , R. H. Dinegar , J. Am. Chem. Soc. 1950, 72, 4847–4854.

[29] C. B. Whitehead , S. Özkar , R. G. Finke , Chem. Mater. 2019, 31, 7116–7132.

[30] N. D. Loh , S. Sen , M. Bosman , S. F. Tan , J. Zhong , C. A. Nijhuis , P. Král , P. Matsudaira , U. Mirsaidov , Nat. Chem. 2017, 9, 77–82. 27995918 10.1038/nchem.2618

[31] D. Gebauer , A. Völkel , H. Cölfen , Science 2008, 322, 1819–1822. 19095936 10.1126/science.1164271

[32] K. Cao , J. Biskupek , C. T. Stoppiello , R. L. McSweeney , T. W. Chamberlain , Z. Liu , K. Suenaga , S. T. Skowron , E. Besley , A. N. Khlobystov , U. Kaiser , Nat. Chem. 2020, 12, 921–928. 32859955 10.1038/s41557-020-0538-9

[33] R. L. Penn , J. F. Banfield , Science 1998, 281, 969–971. 9703506 10.1126/science.281.5379.969

[34] J. J. D. Yoreo , P. U. P. A. Gilbert , N. A. J. M. Sommerdijk , R. L. Penn , S. Whitelam , D. Joester , H. Zhang , J. D. Rimer , A. Navrotsky , J. F. Banfield , A. F. Wallace , F. M. Michel , F. C. Meldrum , H. Cölfen , P. M. Dove , Science 2015, 349, aaa6760. 26228157 10.1126/science.aaa6760

[35] B. Ni , G. González-Rubio , F. Kirner , S. Zhang , H. Cölfen , Angew. Chem. Int. Ed. 2022, 61, e202200753. 10.1002/anie.202200753 PMC9310755 35238123

[36] G. Wulff , Z. Kristallogr. Cryst. Mater. 1901, 34, 449–530.

[37] M. R. Langille , M. L. Personick , J. Zhang , C. A. Mirkin , J. Am. Chem. Soc. 2012, 134, 14542–14554. 22920241 10.1021/ja305245g

[38] S. K. Meena , M. Sulpizi , Langmuir 2013, 29, 14954–14961. 24224887 10.1021/la403843n

[39] S. K. Meena , M. Sulpizi , Angew. Chem. Int. Ed. 2016, 55, 11960–11964; 10.1002/anie.201604594 27560039

[40] C. J. Johnson , E. Dujardin , S. A. Davis , C. J. Murphy , S. Mann , J. Mater. Chem. 2002, 12, 1765–1770.

[41] T. K. Sau , C. J. Murphy , J. Am. Chem. Soc. 2004, 126, 8648–8649. 15250706 10.1021/ja047846d

[42] S. K. Meena , S. Celiksoy , P. Schäfer , A. Henkel , C. Sönnichsen , M. Sulpizi , Phys. Chem. Chem. Phys. 2016, 18, 13246–13254. 27118188 10.1039/c6cp01076h PMC5159743

[43] H.-L. Wu , C.-H. Kuo , M. H. Huang , Langmuir 2010, 26, 12307–12313. 20557088 10.1021/la1015065

[44] T. Ming , W. Feng , Q. Tang , F. Wang , L. Sun , J. Wang , C. Yan , J. Am. Chem. Soc. 2009, 131, 16350–16351. 19856912 10.1021/ja907549n

[45] J. Zhang , M. R. Langille , M. L. Personick , K. Zhang , S. Li , C. A. Mirkin , J. Am. Chem. Soc. 2010, 132, 14012–14014. 20853848 10.1021/ja106394k

[46] W. Niu , S. Zheng , D. Wang , X. Liu , H. Li , S. Han , J. Chen , Z. Tang , G. Xu , J. Am. Chem. Soc. 2009, 131, 697–703. 19102696 10.1021/ja804115r

[47] F. Kirner , P. Potapov , J. Schultz , J. Geppert , M. Müller , G. González-Rubio , S. Sturm , A. Lubk , E. Sturm , J. Mater. Chem. C 2020, 8, 10844–10851.

[48] Y. Wang , J. He , C. Liu , W. H. Chong , H. Chen , Angew. Chem. Int. Ed. 2015, 54, 2022–2051; 10.1002/anie.201402986 25536948

[49] D. R. Lide , CRC handbook of chemistry and physics, Vol. 85, CRC press, 2004.

[50] F. Meng , S. A. Morin , A. Forticaux , S. Jin , Acc. Chem. Res. 2013, 46, 1616–1626. 23738750 10.1021/ar400003q

[51] B. Ni , X. Wang , Chem. Sci. 2015, 6, 3572–3576. 29511518 10.1039/c5sc00836k PMC5812541

[52] B. Ni , H. Liu , P.-p. Wang , J. He , X. Wang , Nat. Commun. 2015, 6, 8756. 26510862 10.1038/ncomms9756 PMC4640082

[53] J. Xiao , S. Liu , N. Tian , Z.-Y. Zhou , H.-X. Liu , B.-B. Xu , S.-G. Sun , J. Am. Chem. Soc. 2013, 135, 18754–18757. 24299234 10.1021/ja410583b

[54] D. R. Hamilton , R. G. Seidensticker , J. Appl. Phys. 1960, 31, 1165–1168.

[55] M. Sun , Z. Cheng , W. Chen , M. Jones , ACS Nano 2021, 15, 15953–15961. 34554725 10.1021/acsnano.1c04056

[56] B. Wiley , Y. Sun , Y. Xia , Acc. Chem. Res. 2007, 40, 1067–1076. 17616165 10.1021/ar7000974

[57] M. Rycenga , C. M. Cobley , J. Zeng , W. Li , C. H. Moran , Q. Zhang , D. Qin , Y. Xia , Chem. Rev. 2011, 111, 3669–3712. 21395318 10.1021/cr100275d PMC3110991

[58] X. Xia , J. Zeng , B. McDearmon , Y. Zheng , Q. Li , Y. Xia , Angew. Chem. Int. Ed. 2011, 50, 12542–12546; 10.1002/anie.201105200 21913296

[59] Y. Wang , D. Wan , S. Xie , X. Xia , C. Z. Huang , Y. Xia , ACS Nano 2013, 7, 4586–4594. 23631674 10.1021/nn401363e

[60] W. Tong , M. J. Walsh , P. Mulvaney , J. Etheridge , A. M. Funston , J. Phys. Chem. C 2017, 121, 3549–3559.

[61] M. García-Ruiz Juan , E. Melero-García , T. Hyde Stephen , Science 2009, 323, 362–365. 19150841 10.1126/science.1165349

