# Supplementary material for: How a Facet of a Nanocrystal Is Formed: The Concept of the Symmetry Based Kinematic Theory
Source: Chemphyschem. 2022 Nov 9;24(2):e202200480. doi: 10.1002/cphc.202200480 (PMC10098540; doi:10.1002/cphc.202200480)
Supplement: Supplementary file 1 — Supporting Information [file CPHC-24-0-s001.pdf]

ChemPhysChem

Supporting Information

How a Facet of a Nanocrystal Is Formed: The Concept of the Symmetry Based Kinematic Theory

Bing Ni,* Guillermo González-Rubio,* and Helmut Cölfen*

Supporting Information

Further discussion 1: Preferential growth directions (PGDs) and the symmetry elements of the lattice:

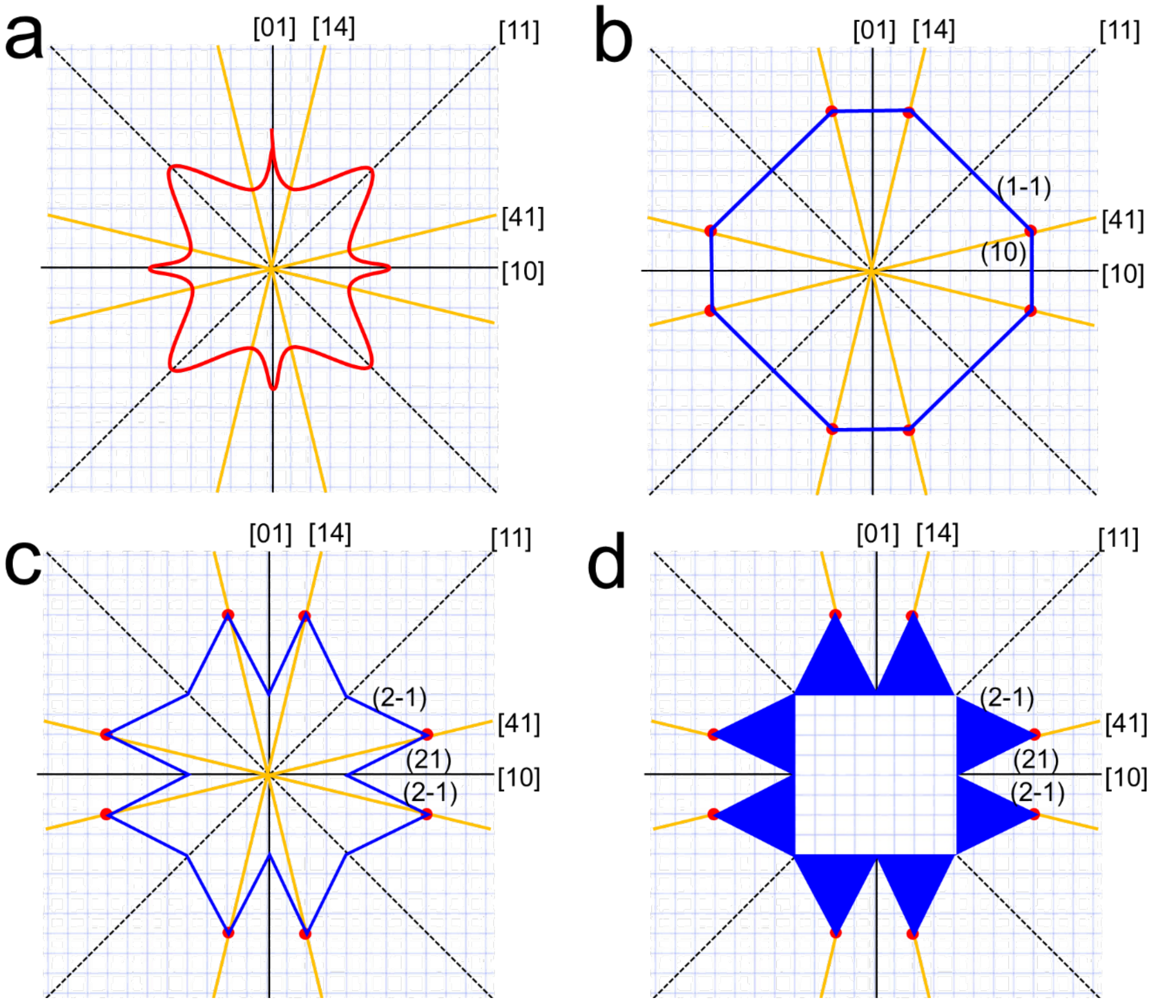

PGDs could guide our prediction of shape evolutions. They are easy to obtain and use. We have already proven that, even though the polar diagram is not known, (1) the growth trajectories of the {01} and {11} facets will always be along their normals, respectively; (2) their growth speed must be the local minimum or maximum. If one set of these directions is the set of directions, which grow globally the fastest, we could easily use these directions to depict the shape evolutions (Figures 4d3, f). Here we will analyze if there exists another global minimal in the slowness vector plots (the global fastest growth directions), as well as their consequences, without considering the physical details about why these directions are the fastest growth directions.

(a) The red line indicates a slowness vector polar diagram with <41> directions (orange lines) as the global fastest growth directions. This is just an example. The same analysis could be extended to any other direction.

(b) If no high index facets exist in the final products, the final shape could be obtained by linking the red dots at equivalent positions of the <41> directions. Therefore, a truncated square would be obtained.

  (1) The truncated square can be also seen as the intermediate structure shown in Figure 4d. In this graph, the truncated square can be obtained by considering the <01> directions as the fastest growth directions. The reason for the formation of truncations could be: i) the fast layer advancement process makes small terraces in the tip domain (Figure 5c); ii) lack of precursors to fully fill the tip terrace;

  (2) If the <41> directions are the fastest growth directions during all the growth processes, the ratio of (1-1) facet area to (10) facet area should keep constant during the whole growth process. If the ratio changes, then the <41> directions are not the fastest growth directions during the whole growth process anymore. However, the ratio between low index facet areas might change during the real growth conditions. Like the cases shown in Figures 2, 3, they could be seen with the fastest

growth directions that are different from the <100> and <111> directions. However, if we want to use directions that really grow the fastest to guide the trajectory prediction, it would be quite difficult to experimentally find the directions.

   (3) Instead, the shape evolutions in these cases could be nicely explained by PGDs of directions along the mirror planes (like <01> or <11> directions).

(c) If high index facets could be obtained, branched structures could be achieved by connecting the equivalent sites, meanwhile the exposed facets should be the same ({21} facet here for example).

   (1) The fastest growth directions (<41> directions) are not necessarily linked with the exposed facets ({21} facets)

   (2) The final structure contains fully equivalent exposed facets. (the (2-1) facet and the (21) facet are crystallographic equivalents, the upper (2-1) facet and the lower (2-1) facet are fully equivalent in any sense, just for example). Therefore, the final structure could be seen as the independent growth of different domains as well, since the building block cannot differentiate the difference between each equivalent domain.

(d) The 8 blue triangles are crystallographically equal. According to the translational symmetry of the crystal, their growth could be seen as independent branching out at different sites of the square seeds, with the branching direction along <01> directions.

Altogether, even if there exist any directions other than those along the mirror planes that grow globally the fastest, the growth trajectory could be obtained by considering those along the mirror planes as the PGDs as well. Since those directions are easy to obtain and use according to the lattice symmetry, we would prefer to use them as the PGDs to understand the growth trajectories.

Further discussion 2: SBKT in other lattices:

Crystal family	5 Bravais lattices		Mirror plane along with the directions	Possible PGDs
	Primitive (p)	Centered (c)		
Monoclinic (m)	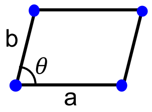 Oblique (mp)		N/A	Need to be studied by cases
Orthorhombic (o)	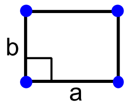 Rectangular (op)	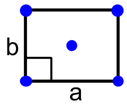 Centered rectangular (oc)	<10>, <01>	<10>, <01>
Tetragonal (t)	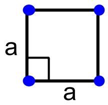 Square (tp)		<10>, <11>	<10>, <11>
Hexagonal (h)	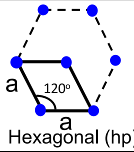 Hexagonal (hp)		<10>, <11>, <21>	<10>, <11>, <21>

The PGDs in other lattices could be obtained from the mirror symmetry analysis as well. Once the PGDs are known, the same thing as shown at Figure 4d can be done to obtain the final structures.

Figure S1: kinematic wave properties:

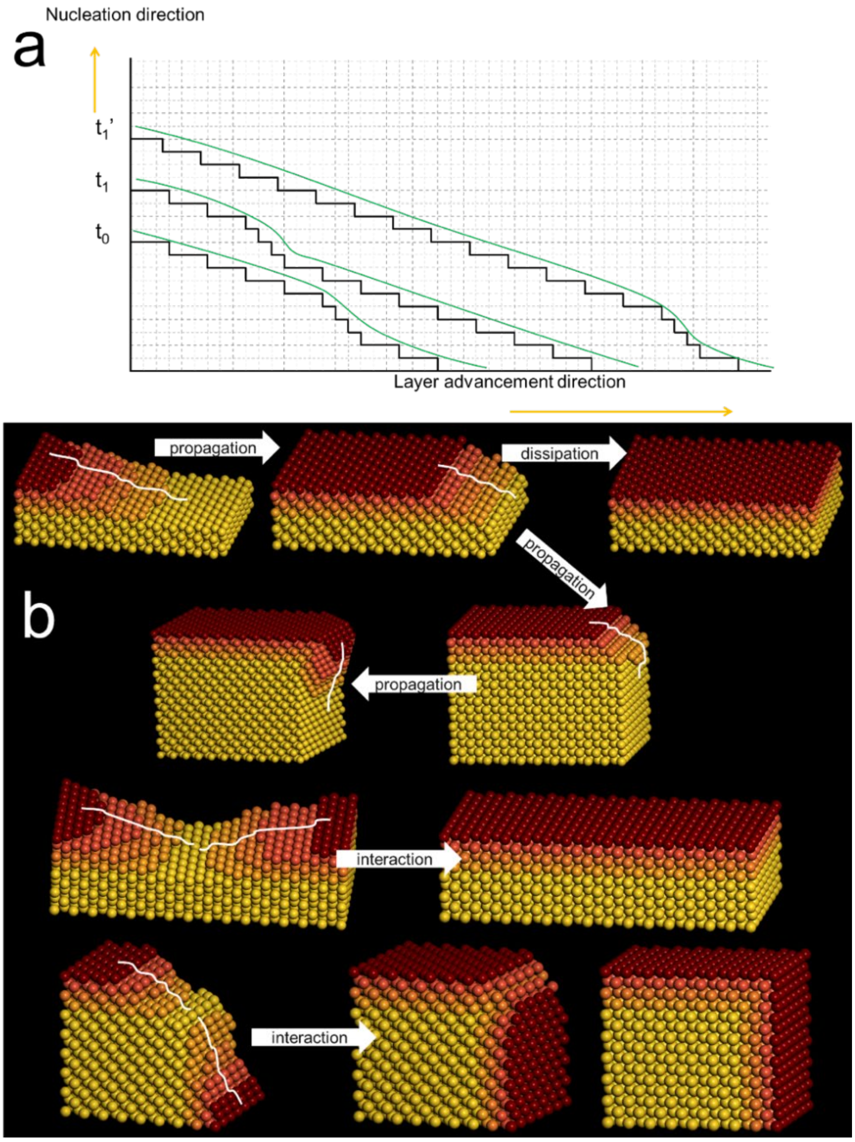

The movement, interactions, and dissipation of kinematic waves

(a) Green lines indicate the outline of the shape. If the one-atom steps move slower than the multi-atom steps, the front step can leave the non-uniformity, and the back step can be caught by the latter multi-atom steps. Accordingly, the non-uniformity would move from the center (t_0) to the left (t_1).

Similarly, if the one-atom steps move faster than the multi-atom steps, the non-uniformity would move from the center (t_0) to the right (t_1').

The kinematic wave is not really a wavy structure. It is the collective movement behavior of the layer advancement. The mathematics of the movements of such a non-uniformity can be described by waves. A detailed analysis is not necessary here.

(b) White lines indicate the terraces, which might be non-uniformities in the structures. The movement of the white lines can be regarded as the kinematic waves.

Reproduced with permission from ref[1]. Copyright 2022 2022 Wiley-VCH.

Figure S2: non-uniformities

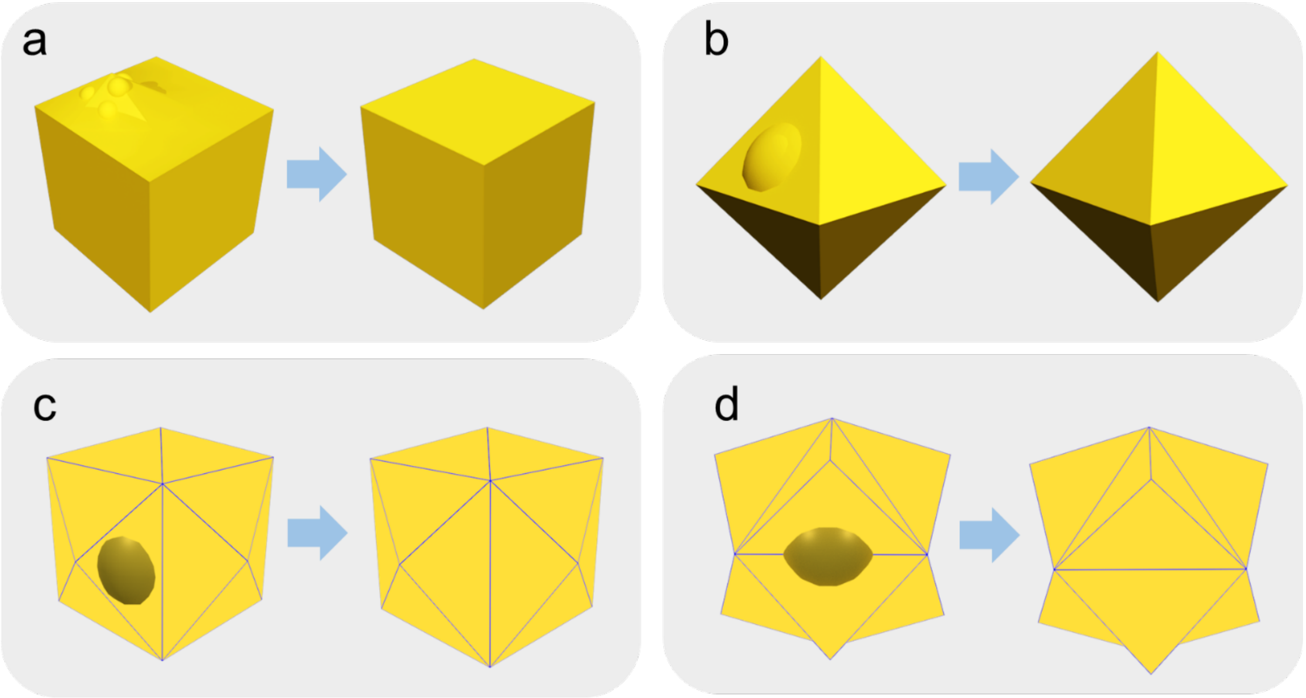

(a, b) protrusions in the cube or octahedron are the non-uniformities, and they would disappear after the growth;

(c, d) The tetrahexahedron and trisoctahedron feature high index facets, and the small spherical protrusions on the surface are also composed of the high index surfaces. However, the formation of these protrusions would break the symmetry of the original nanoparticles (4/m $\bar{3}$ 2/m), therefore they are the non-uniformities.

Figure S3: shape formations in 3D cases by linking the corresponding equivalent points

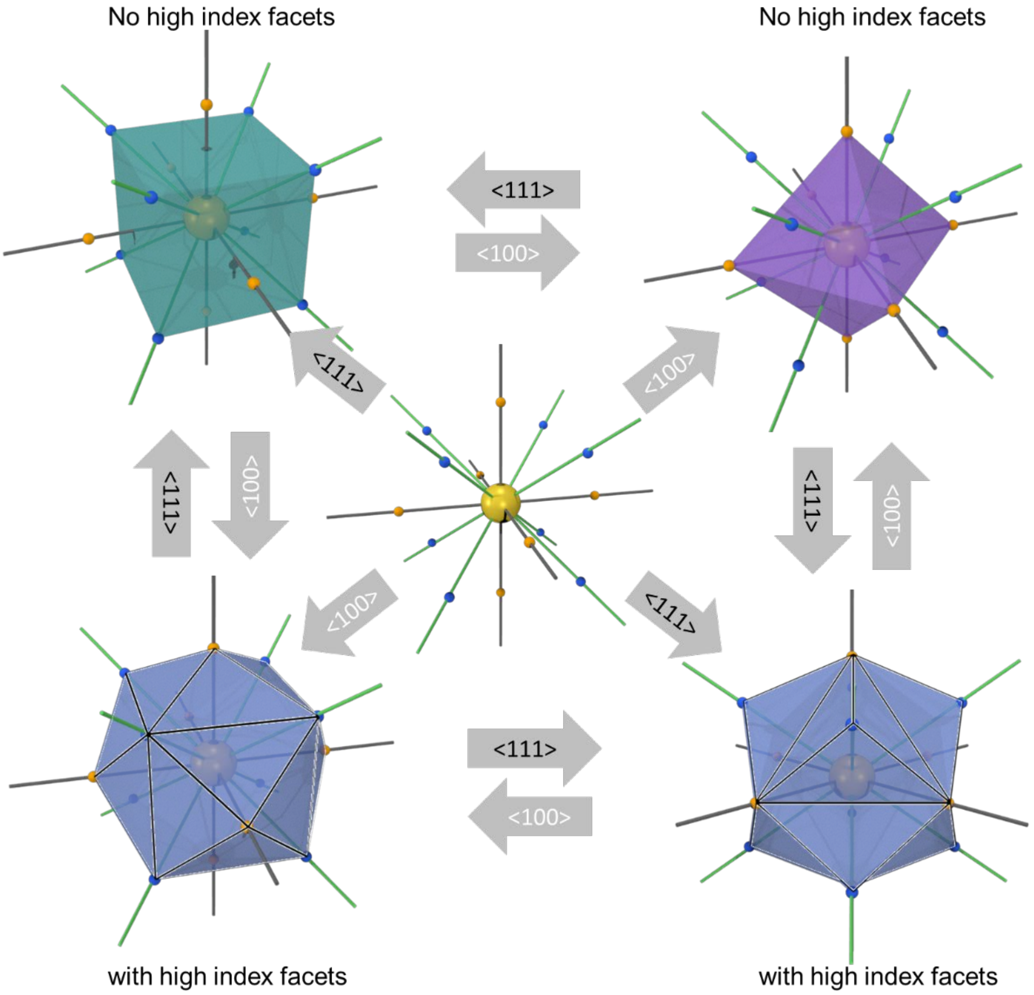

Figure S4: Using the SBKT to explain the shapes shown in Figure 2

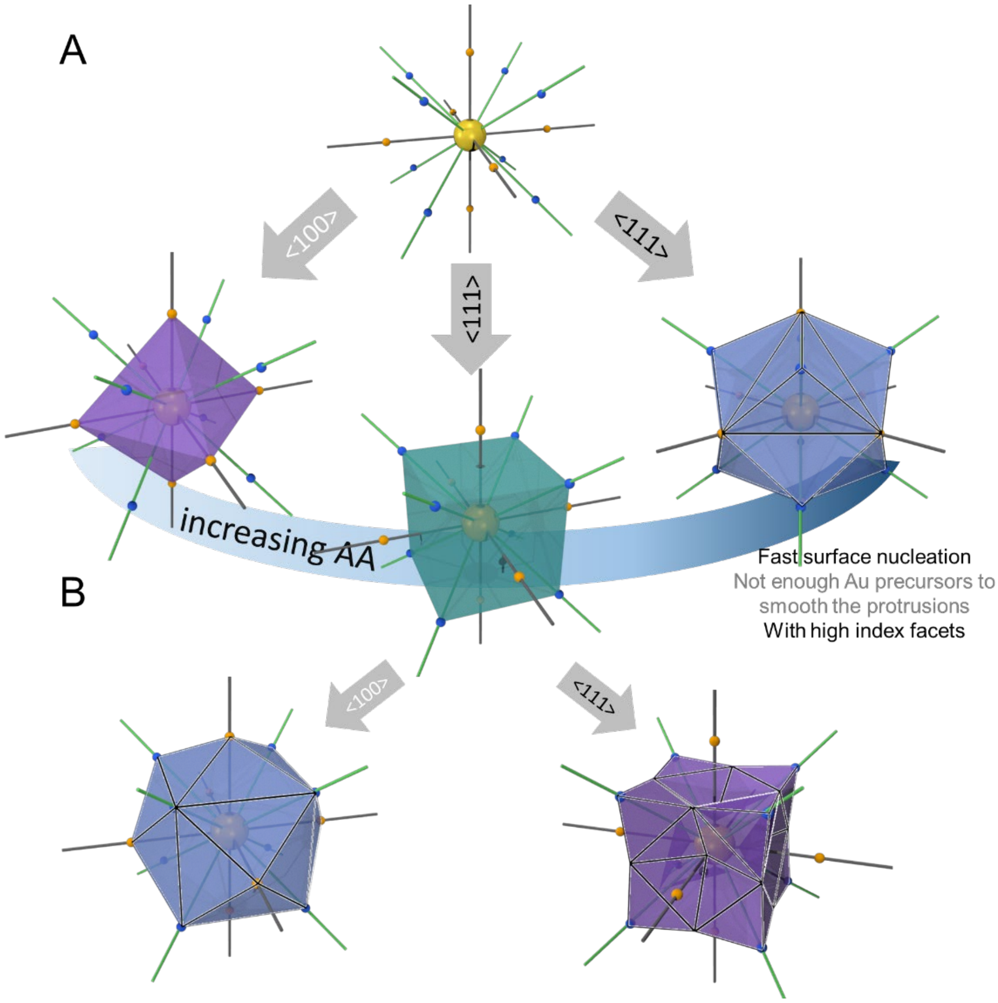

(A) The PGDs could change from <100> to <111> directions with increased AA concentrations. The formation of the trisoctahedron is due to the deficiency of Au precursors. When the consumption of the Au precursor is not extremely fast, cubes would be the main product. However, when the Au precursor is consumed in a very short time due to the very high AA concentrations, trisoctahedra could be obtained since there are not enough Au atoms to fully smooth the tip protrusions (formed by rapid surface nucleation processes) to form a flat cube structure.

(B) Convex or concave structures obtained with different PGDs.

Figure S5: Using the SBKT to explain the shapes shown in Figure 3

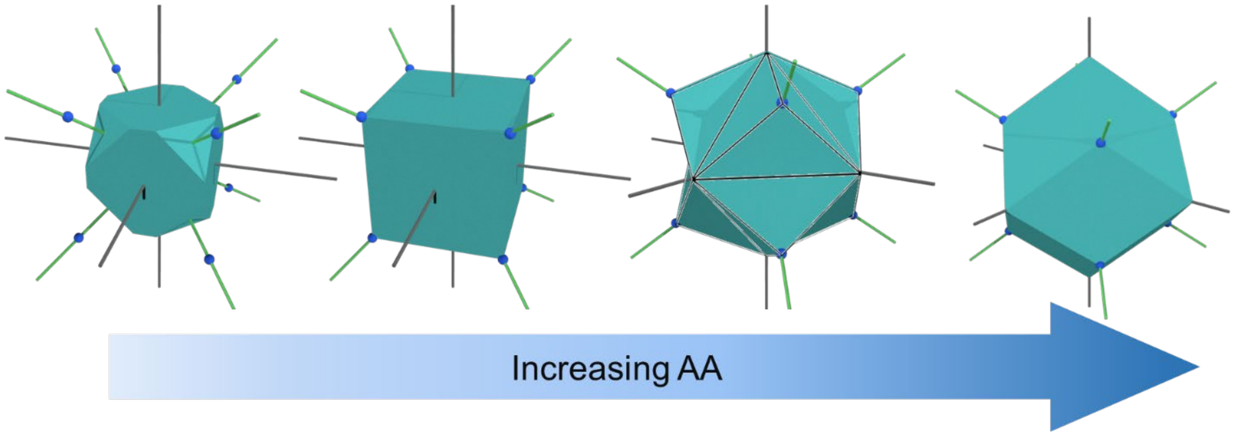

With PGDs of <111> directions, the sizes of the {111} facets would decrease and eventually disappear. When the growth rate is very high and the consumption of Au precursors is very rapid, trisoctahedra could be obtained since there are not enough Au atoms to fully smooth the tip protrusions (formed by rapid surface nucleation processes) to form a flat cube structure. When the layer advancement rate is even faster (layer advancement rate is generally linearly related to the precursor concentrations, while nucleation rate has an exponential relationship to the precursor concentrations. However, nucleation rate is also decided by the size of the nucleation sites. If the size of the nucleation site is too small, the nucleation rates would be greatly hindered due to the low probability of nucleation events[2]), concave sites would be fulfilled and therefore a rhombic dodecahedron is formed.

Figure S6: The shape evolution of Ag nanocrystals reported in literature.

Reproduced with permission from ref[3]. Copyright 2011 Wiley-VCH.

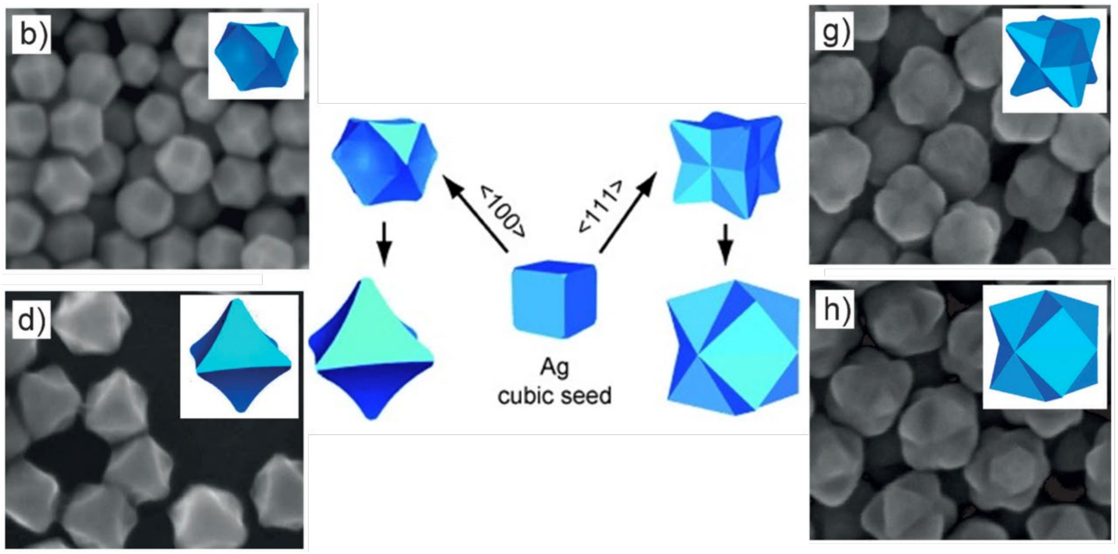

[1] B. Ni, G. González-Rubio, F. Kirner, S. Zhang, H. Cölfen, *Angew. Chem. Int. Ed.* **2022**, *61*, e202200753.

[2] I. V. Markov, *Crystal growth for beginners: fundamentals of nucleation, crystal growth and epitaxy*, World scientific, **2016**.

[3] X. Xia, J. Zeng, B. McDearmon, Y. Zheng, Q. Li, Y. Xia, *Angew. Chem. Int. Ed.* **2011**, *50*, 12542-12546.
